# Remote Monitoring System of Dynamic Compression Bracing to Correct Pectus Carinatum

**DOI:** 10.3390/s23094427

**Published:** 2023-04-30

**Authors:** António Real, Pedro Morais, Bruno Oliveira, Helena R. Torres, João L. Vilaça

**Affiliations:** 1 2Ai—School of Technology, IPCA, 4750-810 Barcelos, Portugal; pmorais@ipca.pt (P.M.); boliveira@ipca.pt (B.O.); htorres@ipca.pt (H.R.T.); jvilaca@ipca.pt (J.L.V.); 2 LASI—Associate Laboratory of Intelligent Systems, 4800-058 Guimaraes, Portugal; 3 Algoritmi Center, School of Engineering, University of Minho, 4800-058 Guimaraes, Portugal; 4 Life and Health Sciences Research Institute (ICVS), School of Medicine, University of Minho, 4710-057 Braga, Portugal; 5 ICVS/3B’s—PT Government Associate Laboratory, 4710-057 Braga/Guimaraes, Portugal

**Keywords:** pectus carinatum, capacitive sensing, magnetometer, pressure, continuous monitoring

## Abstract

Pectus carinatum (PC) is a chest deformity caused by disproportionate growth of the costal cartilages compared with the bony thoracic skeleton, pulling the sternum forwards and leading to its protrusion. Currently, the most common non-invasive treatment is external compressive bracing, by means of an orthosis. While this treatment is widely adopted, the correct magnitude of applied compressive forces remains unknown, leading to suboptimal results. Moreover, the current orthoses are not suitable to monitor the treatment. The purpose of this study is to design a force measuring system that could be directly embedded into an existing PC orthosis without relevant modifications in its construction. For that, inspired by the currently commercially available products where a solid silicone pad is used, three concepts for silicone-based sensors, two capacitive and one magnetic type, are presented and compared. Additionally, a concept of a full pipeline to capture and store the sensor data was researched. Compression tests were conducted on a calibration machine, with forces ranging from 0 N to 300 N. Local evaluation of sensors’ response in different regions was also performed. The three sensors were tested and then compared with the results of a solid silicon pad. One of the capacitive sensors presented an identical response to the solid silicon while the other two either presented poor repeatability or were too stiff, raising concerns for patient comfort. Overall, the proposed system demonstrated its potential to measure and monitor orthosis’s applied forces, corroborating its potential for clinical practice.

## 1. Introduction

Pectus carinatum (PC) is a chest deformity caused by disproportionate growth of the costal cartilages compared with the bony thoracic skeleton, which pulls the sternum forwards, leading to its protrusion, thus resulting in a deformity commonly known as “pigeon chest” [1]. The incidence rate of pectus carinatum is 1:1000 births [2], and is more common in males than females with a ratio of about 4:1 [3]. Typically, this condition does not cause any noticeable symptoms, and people usually seek treatment solely for cosmetic purposes. However, if symptoms do arise, they typically manifest as tenderness at the location of the bulge [4]. In more severe cases where the chest is less flexible, symptoms such as shortness of breath, rapid breathing during physical activity, and reduced stamina may occur [5]. The two main treatment options for this condition are surgical procedures and the use of compressive braces. Typically, for younger patients, braces are preferred due to their less invasive nature and the increased flexibility of the chest wall. On the other hand, surgery is usually recommended for older patients, as their chest walls tend to be stiffer, making the brace exert excessive force which could potentially result in skin complications [6]. Surgery is also recommended in cases of moderate-to-severe asymmetry of the chest wall.

Invasive surgery was primarily proposed as a treatment; however, its long-term effects were inconsistent, frequently resulting in worsened cosmetic appearance, mild protrusion, hypertrophic scarring, skin ulcers, and other postoperative complications [7]. Thus, alternative non-invasive options, namely external compressive bracing applied by orthoses, has become the primary treatment. Currently, the orthoses for bracing consist of one or more aluminium/steel bars, a cushioned pad, and back and shoulder straps. Some examples of commercially available orthoses include the Dynamic Compression System (Pampamed SRL, Buenos Aires, Argentina) [8] and i3DCarinatum (iSurgical3D, Guimarães, Portugal) solutions. This type of orthosis has demonstrated its good results in recent years [9,10]. Clinical guidelines recommend long-treatment periods daily with the standard orthoses, ~23 h/per days, throughout a full period of 4–6 months on average [11]. In this sense, particular attention at the interface structure between the orthoses and the patient chest is mandatory. This structure must be biocompatible, comfortable, thin, breathable, lightweight, sterilizable and washable. Among different options, silicone was explored and integrated in some of the commercial solutions.

However, it has been reported that a large number of patients do not follow the clinically prescribed treatment (e.g., applied pressure, usage time) [12]. Moreover, the systems are blind, not monitoring the applied pressure. While too much pressure can cause pain and pressure ulcers in the patient, inadequate pressure will prolong treatment time and will result in a sub-optimal outcome and consequently some loss of confidence in the treatment outcome. To mitigate the abovementioned limitations, specific researchers studied the integration of sensors to continuously monitor the treatment. Nevertheless, most of the proposed solutions rely on load cells, which drastically increase the dimensions of the orthoses and are uncomfortable. Moreover, the available solutions are focused on the monitoring of pressure at specific temporal moments, namely the clinical evaluations, failing to continuously monitor the treatment.

In this study, we propose a novel strategy to continuously monitor the PC orthosis treatment application. Different constraints were considered at this development: (i) it must allow accurate and precise measurements; (ii) it must not require an increase in the orthoses dimension and weight; (iii) it must be of simple construction/production; and (iv) it must keep the abovementioned requirements for the orthoses-body interface. Thus, a strategy to embed a sensor system into the previously used silicone-interface was studied. Different sensor types were explored, namely: capacitive (with two construction strategies) and magnetic-based (with one construction approach) systems. The accuracy of the developed systems was used in simulation and controlled experimental studies. Moreover, the initial concept of full architecture for in-site signal processing and communication and an intuitive software for home-monitoring are described.

Overall, the current work introduces the following novelties:

- A new pipeline to produce instrumented PC orthoses by directly embedding the electronic devices into the silicone interface.
- A comparison study of silicone embedded capacitive and magnetic sensors.
- The initial concept of a full framework for home-monitoring of the treatment.

The remaining article is structured as follows. In Section 2, a technical description of the silicone-embedded sensor and the monitoring framework is presented. Section 3 present the validation experiments that were performed and associated results, respectively. Section 4 discusses the performance of the proposed pipeline. Finally, the conclusions are pointed out in Section 5.

## 2. Materials and Methods

**Figure 1:**
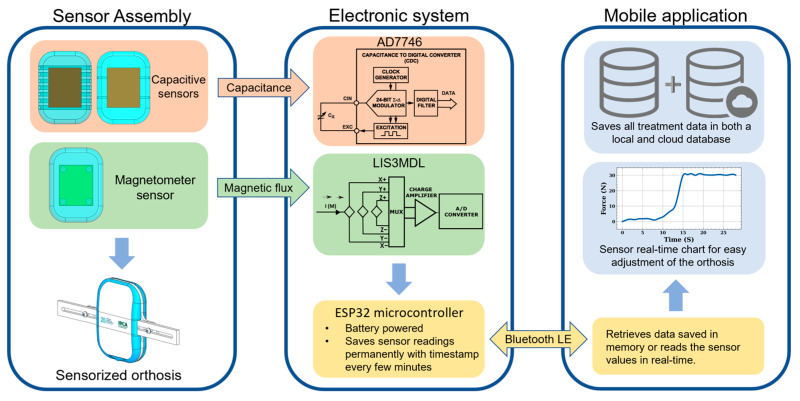
Overview of the different components of the proposed system.

In this section, a detailed explanation of the full pipeline to monitor PC orthoses treatment is presented (see Figure 1). Due to its importance for the entire system, particular emphasis on the silicone-embedded sensor construction is explored. Two sensor’s types are investigated, namely the capacitive, describing two construction strategies (Section 2.1 and Section 2.2), and magnetic ones, with one construction approach (Section 2.3). Finally, in Section 2.4, an initial concept of a framework to allow home-monitoring of the system is presented.

### 2.1. Capacitive Sensor

These sensors are based on capacitors of parallel plates, whose capacitance (C in Faradays [F]) are calculated as [13]:

(1) $$C=\frac{\varepsilon *A}{d},$$

where *ε* is the permittivity (unity F/meter [m]), A the area (in m^2^) and d the distance (in m) between plates.

For a well-known structure (i.e., known area and ε), the variation of the capacitance is directly related to the modification of the distance between parallel plates, simplifying Equation (1) to:

(2) $$C\propto \;\frac{1}{d},$$

Note that the modification of the distance between plates can be related to external forces or pressures. Based on this concept, two different construction types are proposed, namely: (1) polyurethane (PU) foam based capacitive sensor and (2) perforated silicone-based capacitive sensor. For each sensor, the capacitance of the system is measured. The variations of the capacitance are used to infer the pressure through a characteristic curve obtained in compressions tests (see Section 3).

#### 2.1.1. PU Foam-Based Capacitive Sensor

**Figure 2:**
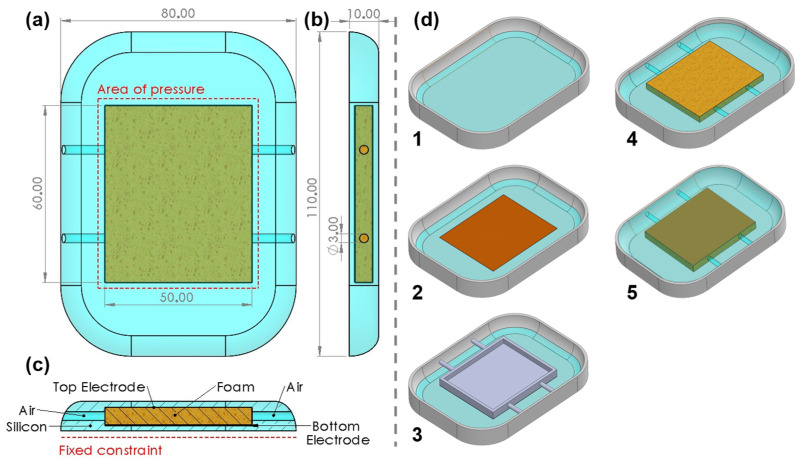
PU Foam capacitive sensor, (**a**) Front view of sensor, electrode area outlined, (**b**) right side view of sensor, (**c**) bottom view of sensor (back of sensor is fixed constraint in compression tests), (**d**) step sequence of sensor construction. All dimensions are in millimeters.

For the construction of this sensor (see Figure 2), firstly a mold was designed using the computer-aided design software Solidworks^®^ (SolidWorks Corp., Concord, MA, USA) with the outer shape and dimensions of one commercial PC treatment solution, as shown in Figure 2(d1). Then, a thin layer of silicon is poured and dried, the electrodes and a flexible structure are placed on top, as shown in Figure 2(d4), and then silicon is poured again until it fills the mold. The flexible structure relies on a PU foam, which is used to magnify the variation between sensors plates when external pressure is applied. Since the PU foam is porous and holds air, four channels were made in the silicon by using an auxiliary mold (Figure 2(d3)). Thus, the air can move outside or inside of the structure as the sensor is pressed, thus increasing the sensitivity of the sensor.

#### 2.1.2. Perforated Silicon-Based

**Figure 3:**
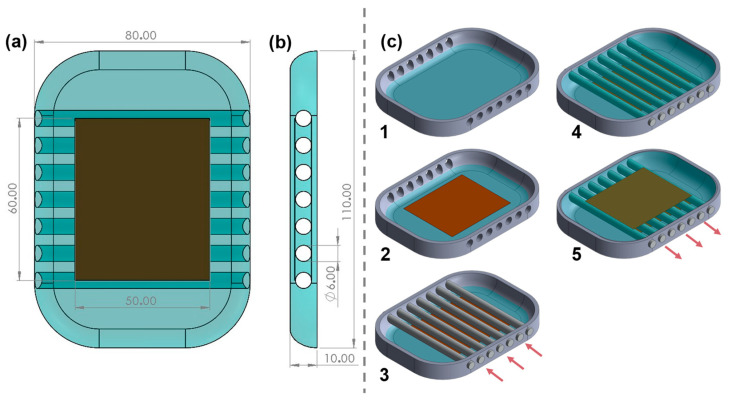
Perforated silicone capacitive sensor: (**a**) front view of sensor, (**b**) right side view of sensor, (**c**) step sequence of sensor construction. All dimensions in millimeters.

Instead of foams, we also researched the potential of a simpler construction approach, entirely based on silicone and electrodes. As an initial concept, the mechanical properties of a solid silicone structure (including silicone between electrodes) were studied. Secondly, a second strategy based on perforated silicone was proposed. The perforated silicone reduces the rigidity of the structure, increasing the sensibility of the sensor to small displacement. The sensor is constructed according to the process shown in Figure 3(c1). In summary, to create the holes (detailed in Figure 3), we modified the exterior mould, creating circular entry points where aluminium rods (chosen for their smooth surface) can be inserted, as represented in Figure 3(c3) by the arrows. Silicone is then poured into the mould and after curing, the rods are removed by pushing them aside in the arrows’ direction, as seen in Figure 3(c5), leaving the circular holes in the silicone. By placing holes in the silicon, empty interfaces are created in the structure (see Figure 3b), which will facilitate displacement between sensors (when compared with a compact silicone structure), thus increasing its sensibility.

### 2.2. Magnetometer-Based Sensor

**Figure 4:**
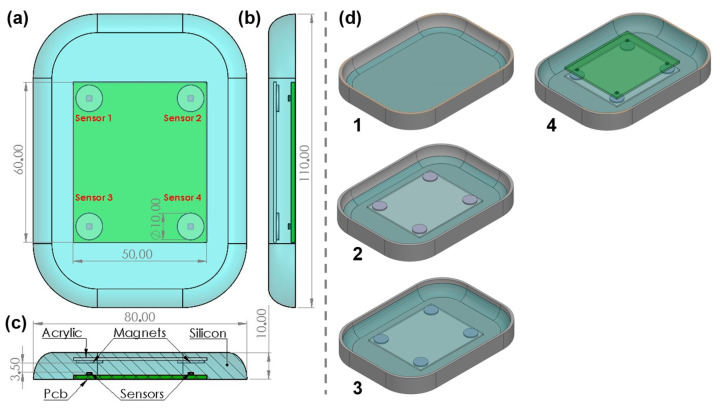
Magnetic sensor structure: (**a**) front view of sensor, (**b**) right side view of sensor, (**c**) section cut detailing all layers and materials, (**d**) step sequence of sensor construction. All dimensions in millimeters.

Differently, three-axis magnetometers were also researched to construct the silicone-embedded sensor [14]. The magnetometer measures the strength and direction of the magnetic field in the neighborhood of the sensor by decomposing the magnetic field reading in three components according to the coordinate system of the sensor. Based on this concept, a grid of magnetometers was created to locally monitor the applied pressure (see Figure 4). For each sensor, a magnet was positioned in a thin acrylic rectangle, which was connected to the orthoses. The magnet was aligned with the *z*-axis of the magnetometer, with a fixed offset of 3.5 mm. Empirical studies were performed to determine the offset, i.e., a non-saturation region. To construct this sensor, the following method was followed (see Figure 4d): pour a thin layer of silicone and then place the acrylic piece with the magnets glued to its corners. Pour silicon until nearly the top, leaving a small gap to place the printed circuit board (PCB) that contains the sensors and let it dry. Finally, the PCB is placed on top of the silicon and the gaps around the PCB are filled until the top of the mold.

Therefore, the application of pressure will result in a displacement of the magnets and consequently a modification of the measured magnetic field. Only the Z-component of the magnetic field is monitored. To infer the applied pressure, a characterization of the sensor in a compression study was performed (see Section 3).

### 2.3. Implementation Details

Concerning the sensor materials/components, it consists of flexible silicon (HB5513, HBQuimica, Porto, Portugal), polyurethane (PU) foam (2350LA, Euroespuma, Espinho, Portugal) and two copper tape electrodes. The silicone material presents a Young’s modulus of approximately 720 kPa, linear contraction < 0.05% and stretching until breaking of 450%. For the magnetometer, 4 LIS3MDL (STMicroelectronics, Geneva, Switzerland) were used.

### 2.4. Monitoring Framework

**Figure 5:**
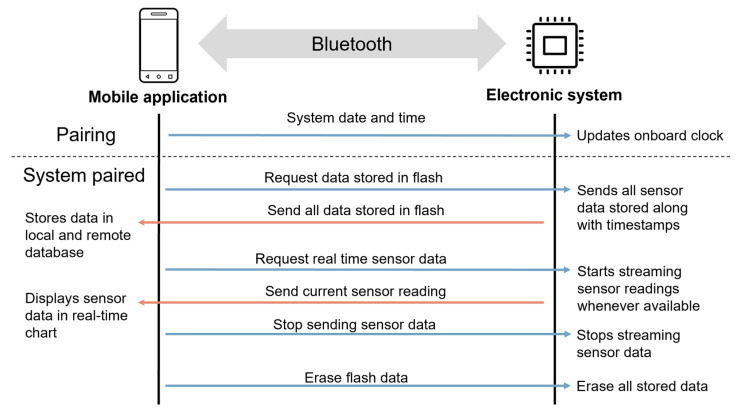
Overview of data communication, processing and visualization pipeline detailing how the mobile application and electronic system interact.

To allow simple access to the developed embedded-silicone concept, data communication, processing and visualization pipeline based on a mobile application is next presented (Figure 5).

#### 2.4.1. Signal Acquisition and Processing System

Concerning the electronic system, the following requirements were considered: (i) it must be compact, (ii) be capable of permanently saving the values of the sensors; (iii) powered by a battery (thus having low power consumption); and, (iv) capable of wirelessly transmitting the saved data whenever requested. Considering the remaining requirements, the following components were chosen:

- ESP32-WROOM-32, a microcontroller with embedded flash memory to permanently store data and capable of Bluetooth Low Energy (BLE) communication to transmit saved data, inexpensive when compared to other modules. It has the advantage of being a pre-certified module by the Federal Communications Commission (FCC).
- AD7746, a 24-bit resolution capacitance to digital converter, responsible for acquiring capacitive sensor readings up to 21 pF of bulk capacitance plus 4 pF of changing capacitance.
- DS3231M, a real-time clock (RTC) to keep track of the time and date of when each sensor reading is registered.
- MCP73831, responsible for managing the charging the lithium polymer battery.
- APA102, an addressable RGB led to provide visual feedback to the user, turns on with red color to indicate when BLE is on and blue when to phone establishes a connection.
- LIS3MDL, returns the XYZ magnetic field of a nearby magnet relative to the sensor itself. These sensors have 16-bit data output and have ±4/±8/±12/±16 gauss selectable magnetic full scales for all axes. In this case, ±4 gauss was chosen as this range provides more precise measurements and is adequate for the used magnets. These sensors require an auxiliary PCB to be placed under the silicon pad.

The microcontroller was programmed to save a sensor reading every 30 min (configurable) and it must be in a deep sleep state during the remaining time to minimize power consumption. A total of 64 thousand entries can be saved in the flash memory, so it would take approximately 3.5 years of saving data to run out of memory, which far exceeds the treatment period. This way, the patient only has to retrieve data periodically. For user interaction/feedback, it has two buttons, one for resetting and one for turning on BLE. The RGB LED indicates BLE state, red when merely powered and blue when paired with the phone’s mobile app. An orange LED is turned on when charging the battery (micro-USB cable plugged to a wall adapter e.g., a phone charger) which then turns off when the battery is fully charged. Power consumption was measured at 0.5 mA when in deep sleep, 80 mA when saving sensor readings in flash memory and 130 mA when transmitting data using BLE. For example, if a 1000 mAh lithium-ion polymer battery is used, battery life is estimated to be approximately 20 days, and a full recharge takes 2 h.

#### 2.4.2. Mobile Application

To allow home-monitoring of the system, a mobile application (compatible with Android and IOS) was created. It was developed using Qt (The Qt Company, Espoo, Finland) and has the following features:

- Ability to pair and communicate with the embedded system using the BLE protocol.
- Synchronize RTC’s date and time with the phone’s upon pairing to guarantee the RTC is always up to date.
- Visualization of sensor data in real-time, useful for the patient to adjust the orthosis to the correct pressure.
- Retrieve and delete data saved in the embedded system flash memory which in turn is permanently saved in the application in both a local and online database.

### 2.5. Experimental Setup

**Figure 6:**
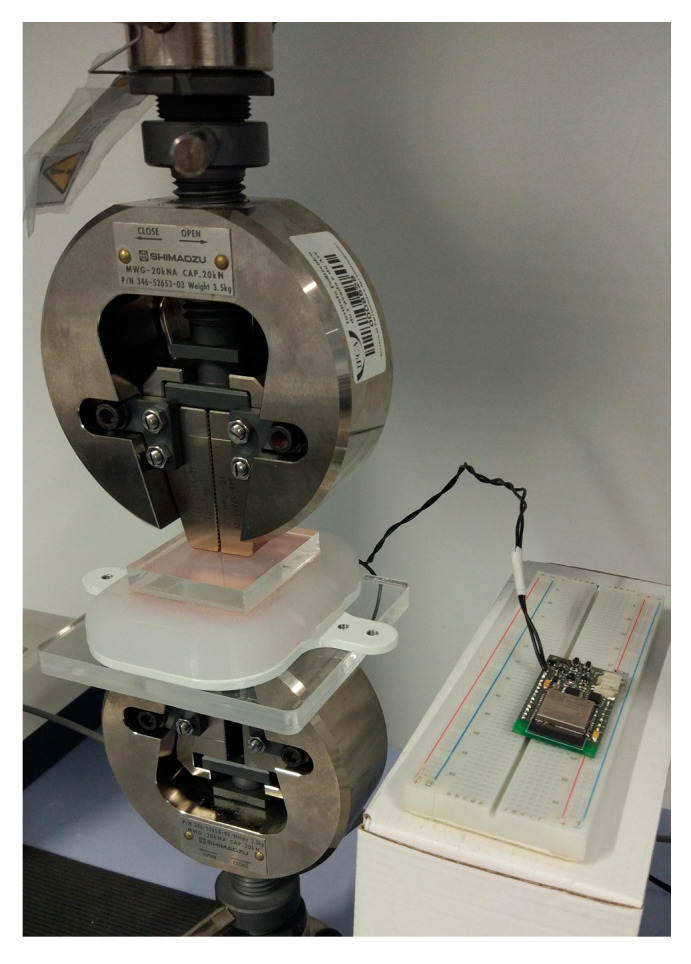
Load cell applying pressure to PU foam sensor.

To measure the accuracy of each sensor, two experiments were carried out using a compression testing machine (Figure 6) with a load cell of 10 kN (AGS-X, Shimadzu, Kyoto, Japan): (a) a global compression test—to evaluate the behaviour of the sensor when strength is applied to all sensor surface; (b) a local compression test—to evaluate the behaviour of the sensor when strength is applied to a part of the sensor surface.

The capacitive sensors’ response variation with the temperature was measured. Finally, the power consumption of the system was measured using a current sense shunt resistor and an oscilloscope to see the current waveform. From the waveform, an average value was registered for different situations: circuit in deep sleep, with BLE on, saving values in flash memory.

#### 2.5.1. Global Compression Test

We modified the applied force by the calibration machine, ranging from 0 N to 300 N at 1 mm/s, across the total area of the sensor (red outline Figure 2a) and we collected the sensor response (i.e., the capacitance or magnetic field). The range of forces applied to the sensor was chosen according to [15].

In total, 10 data points were collected, once every 30 N and for each data point. For each data points, 10 readings of the sensor were registered and averaged. These compression tests were repeated five times to verify the repeatability of the results.

#### 2.5.2. Local Compression Test

**Figure 7:**
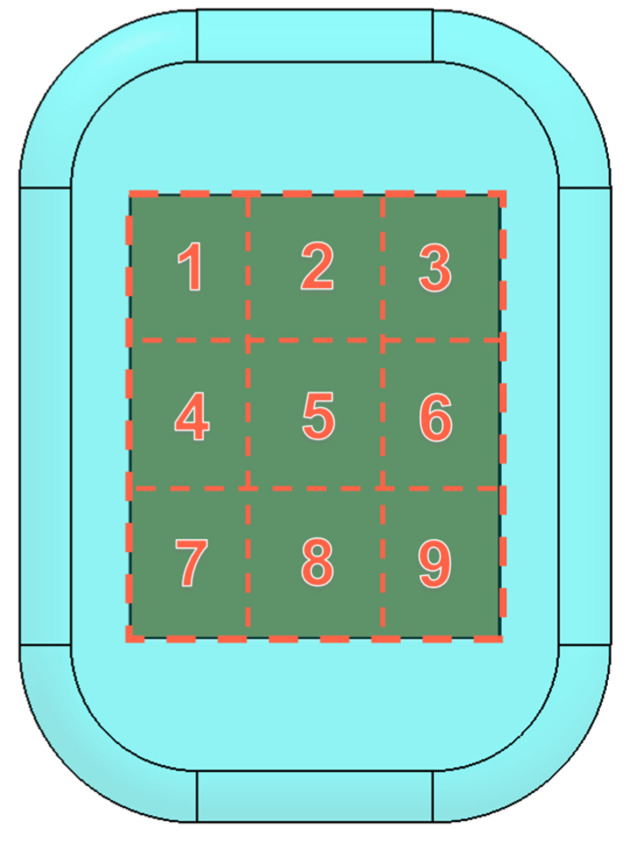
Division of sensor in smaller regions where pressure was applied, numbered 1 to 9.

Embedding materials in the silicone pad or changing its geometry can have consequences such as making it stiffer, possibly making it uncomfortable for wearing. Thus, a last experiment was conducted to locally evaluate how embedding materials in the silicone pad affects its mechanical response. In this sense, each sensor was divided into nine smaller areas and the pressure was applied independently in each area (Figure 7). To apply the same pressure on the smaller areas, the pressure was calculated through $\sigma =\frac{F}{A}$, where *σ* is the compressive stress, *F* (in Newton N) is the maximal force and *A* (in mm^2^) is the surface area where pressure is applied. Thus, considering the maximal force of 300 N applied to the area of 3000 mm^2^ (i.e., area of each small region) will results in a compressive force of 0.1 MPa. Therefore, on each region of the sensors, 0.1 MPa was applied and the sensor response registered.

#### 2.5.3. Temperature Influence Test

Considering the silicon pad is in contact with the patient’s chest, the silicon will gradually become warmer. An experiment was performed to evaluate if the temperature increase influences the capacitance readings of the capacitive sensors. The sensors were placed on top of a heat plate and the capacitance readings were first registered at room temperature (25 °C) to have a baseline value. After that, the temperature was increased up to 40 °C in 5 °C intervals, while maintaining each temperature for at least 10 min to make sure all the silicon was at the same temperature. The registered capacitance values are then converted to force using the calibration curves obtained from the compression test of each sensor to evaluate the error magnitude. The tests were repeated three times for each sensor and the average and standard deviation values were calculated.

## 3. Results

**Figure 8:**
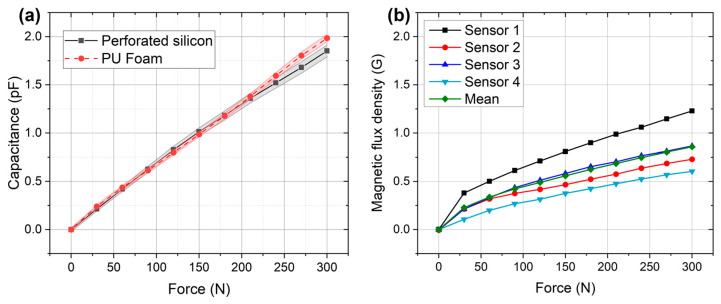
(**a**) Changing capacitance as a function of compressive force for both capacitive sensors, filled area represents standard deviation; (**b**) magnetic flux density measured on the *Z* axis as a function of force on magnetometers.

Figure 8a depicts the result of the experimental tests using load cells. For both situations, the capacitance as measured as a function of compressive forces. Overall, both graphs have high linearity (R^2^ > 0.99). Figure 8b shows a plot of magnetic field strength measured on the *Z*-axis of each magnetometer as a function of the applied compressive forces through the load cell.

**Figure 9:**
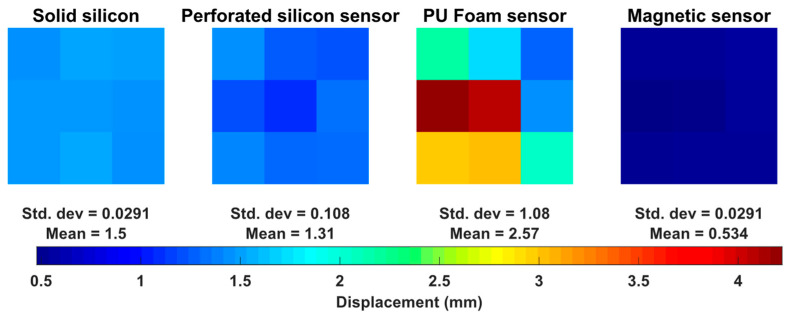
Displacement of sensor regions of the three proposed sensors and a solid silicon pad for comparison.

Concerning the local compression experiments, Figure 9 shows the displacement registered for each of 9 regions considered in this study. The results are presented in an image with 3 × 3 pixels, each pixel represent the evaluated region. The colours represent the displacement in each region when 0.1 MPa of pressure is applied. The global mean and standard deviation value of all 9 regions was also computed.

**Figure 10:**
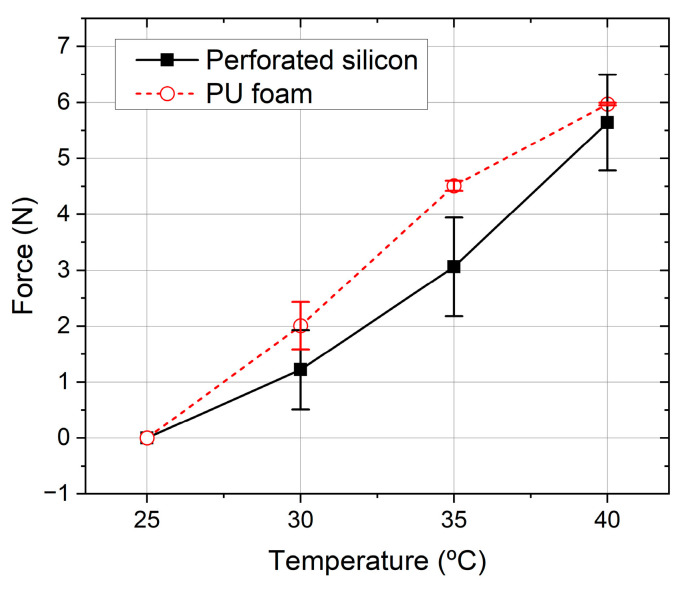
Changes in measured force due to temperature increase in both unloaded sensors. The lines represent the average data of the three tests and the error bars represent the standard deviation for each point.

Figure 10 shows the changes in capacitance with the temperature. If the capacitance errors are converted to force by using the linear regression taken from the Figure 8a plots, the maximum error was 5.97 N for the perforated sensor and 5.64 N for the PU foam sensor.

## 4. Discussion

The concept of monitoring the entire treatment period of PC was explored in [16], where an array of seven pressure sensors was used along with an electronic system for storing of sensor data. The data can be later access by the physician with a custom computer software. In a more recent clinical study [17], the researchers suggested continuous monitoring of the patient’s temperature, inferring the treatment period from the obtained data. Here, mobile applications were released to easily access the data. Nevertheless, several limitations can be mentioned in the previous studies. The first approach [16] required a modification of the orthosis design, increasing its dimension and thickness and consequently making it not practical for routinely use. Additionally, it was not suitable for home-monitoring since no user-friendly interface was developed (only released for medical professionals). Conversely, the second approach [17] was designed to allow continuous monitoring of the treatment by simple inferring the usage period for the PC orthoses. However, the obtained information is limited (only the usage time), failing to capture the applied pressure by the orthosis. Indeed, the monitoring of the applied pressure by the orthosis is a relevant clinical indicator to guarantee optimal outcome of the treatment.

In this study, a new concept for monitoring of the pressure applied by the standard PC orthosis was proposed. Overall, four strategies to construct accurate silicone-embedded sensors without relevant modification of the standard construction processes and without mechanically modifying the commercially available orthoses was presented. The sensors showed its high accuracy for the treatment (Figure 8). Moreover, to promote home-monitoring of the treatment, a simple and user-interface mobile application to access and store the sensor information was developed. Note that the mobile application is equipped with a set of periodic alert messages, remembering the user to perform the treatment monitoring. Additionally, all data are also in an online database, where the clinical teams can access the patient-specific data throughout the full treatment.

### 4.1. Result Analysis

By evaluating the results of both capacitive sensor options (Figure 8a), we could observe that both present a linear response (except at the low-pressure ranges), which makes the calibration of the sensor easier to implement in the firmware of the microcontroller responsible for collecting data and ultimately making it interesting for clinical application for the target pathology. Looking to the slope of each curve, it is possible to observe a high slope in the foam-based approach, presenting therefore a higher sensibility. This difference is as expected, considering the PU foam is still noticeably easier to compress than the perforated silicon. Concerning the magnetic sensors, each magnetic sensor also presented a linear response while increasing the applied force (Figure 8b), with a steeper slope between 0 N and 30 N. This steeper slope can be explained by air bubbles trapped between the acrylic and the silicone, causing the different slope until the silicone is fully compressed after 30 N. Although small differences were observed between sensors, it can be explained by the construction process, and small errors when positioning the magnet in relation to the sensor.

In the second experiment (i.e., regional evaluation), the mean displacement value of the silicon sensor was lower than the PU foam sensor. However, when looking for each region individually, it is possible to observe a variation in the measured value in each portion for the PU foam approach which can be explained by the sensors’ construction. Looking at the cavity where the PU foam resides, it is expected that when applying force in the center will cause a different displacement compared to the edges of the foam. On the other hand, the perforated silicone showed an (approximately) homogenous response, indicating a higher reproducibility and feasibility of this approach. This analysis is relevant in PC, where the patient chest deformity could not be completely aligned and distributed with the sensor centre and area. Thus, our current local analysis demonstrates that the proposed method is feasible in cases of asymmetric PC, while the PU foam sensor would require more care in its placement on the center of the chest bulge. Regarding the magnetic sensor, the displacements across the regions are homogenous but limited, which may affect how comfortable the silicon pad is for the patient.

By evaluating the results of the temperature experiment, it can be observed that the capacitance of both sensors increases with the temperature in an approximately linear fashion. When comparing with the sensor’s full range of 300 N, the reported maximum error of each sensor at 40 °C represents 1.88% and 1.99% for the perforated sensor and the PU foam sensor, respectively. These errors, while considered small, can be further improved in a future work by using the AD7746’s internal temperature sensor to compensate for temperature changes. Concerning the magnetometer-based sensor, it was verified that the magnetic flux density changes for the tested temperature range are within the noise range of the sensor, and therefore were considered negligible.

### 4.2. System Perfomance

The results also demonstrated that by taking into consideration the required force range for the correction of the PC, the sensor can measure such forces with adequate accuracy and sensibility. Moreover, considering its power consumption, the system is ready for continuous monitoring of the treatment, and therefore to improve the current clinical practice. Thus, sending the data to an online database is especially important for the clinician, since non-compliance is one of the most important factors contributing to treatment effectiveness. The disparity between the patient-reported compliance and the data saved by the monitoring system will be more easily evaluated, considering one crucial factor for a successful treatment is patient compliance [18].

The system has the advantages of being easily integrated into the existing orthosis with minimal size increase and the sensors having good linear responses within the chosen range of forces. Moreover, no relevant modification to the current construction strategy is required, easing its integration in the current commercial solution. As an improvement to the current clinical practice, and thanks to the online monitoring and storing available at the proposed system, new clinical indicators can be possibly discovered with this device, and improved medical treatment (by accurately evaluating the pathology evaluation throughout its entire period and not only at clinical consultations) can be provided to the patient.

### 4.3. Limitations and Future Work

Some limitations in the designed sensors should be emphasized:

- Magnetic sensors’ readings are affected by large ferromagnetic objects nearby the orthosis since these alter the shape of the magnetic fields of the magnets.
- Acrylic piece in magnetic sensor drastically diminishes compressibility of silicon the silicon pad, raising concern regarding comfort of the patient.

In a future work, clinical validation of the proposed prototype as well as dedicated clinical trials to exploit all potential of the developed technology are envisioned. Other improvements to the system could also be made. Since compliance is harder especially on young children, one possibly important feature to add in the future would be reward system, where points are rewarded by interacting with the mobile app and showing overall progress of the treatment, in an effort to encourage the use of the orthosis.

## 5. Conclusions

The goal of this study was to develop a system for measuring pressures applied by the orthosis on the PC, as well as, continuing monitoring the treatment. In-vitro studies demonstrated that all three sensors show a linear response while increasing the applied force. The foam-based sensor showed high sensibility, however, it presented lower reproducibility in regional analysis. To correctly test the feasibility of the proposed devices, a clinical study is expected to be conducted in PC patients during the next months. The system and sensors also have the potential to expand into other orthotic and prosthetic devices that require force management.

## Data Availability

Not applicable.

## Abbreviations

The following abbreviations are used in this manuscript:

PC	Pectus Carinatum
PCB	Printed Circuit Board
PU	Polyurethane
